# Effects of intermittent feeding versus continuous feeding on enteral nutrition tolerance in critically ill patients: A protocol for systematic review and meta-analysis

**DOI:** 10.1097/MD.0000000000023528

**Published:** 2020-12-11

**Authors:** Yuanli Li, Jin Yang, Shunxia Sun, Juan Huang, Aiguo Zhang, Xiaoling Tang

**Affiliations:** Department of Critical Care Unit, Chongqing General Hospital, Chomgqing, China.

**Keywords:** continuous feeding, enteral nutrition, intensive care unit, intermittent feeding, meta-analysis, protocol, tolerance

## Abstract

**Background:**

Nutritional support is an indispensable treatment for critically ill patients. Enteral nutrition intolerance is one of the obstacles to the smooth progress of enteral nutrition.

Enteral nutrition can be divided into continuous feeding and intermittent feeding. However, the effectiveness and safety of the 2 ways of nutrition infusion are controversial clinically. Therefore, this meta-analysis further evaluated the effect of intermittent feeding versus continuous feeding on enteral nutrition tolerance in critically ill patients.

**Methods:**

Cochrane Library, PubMed, Web of Science, EMbase, China Biology Medicine disc (CBM), China Science and Technology Journal Database (VIP), China Journal full-text Database (CNKI), and Wanfang Database were searched for all randomized controlled trials (RCTs) on the effects of intermittent and continuous feeding on enteral nutrition tolerance in critically ill patients. The quality of literatures was strictly evaluated and the data were extracted by 2 investigators. Meta-analysis was carried out by applying RevMan 5.5 software.

**Results:**

The results of this meta-analysis are published in peer-reviewed journals.

**Conclusions:**

This study provides reliable evidence-based support for the effects of intermittent and continuous feeding on enteral nutrition tolerance in critically ill patients.

**OSF Registration number:**

DOI 10.17605/OSF.IO/4BP5X

## 1 Introduction

Nutritional support is one of the indispensable ways for human body to obtain nutrition. At present, enteral nutrition is the first option of nutritional support for critically ill patients with intestinal function.^[1,2]^ Enteral nutrition can enhance the immune function of critically ill patients, improve nutritional status, reduce infection rate, shorten hospital stay, and reduce mortality.^[3,4]^ However, enteral nutrition intolerance is one of the obstacles to the smooth progress of enteral nutrition. The average incidence rate is 33%. Especially, the incidence of intensive care unit patients with mechanical ventilation is as high as 80.2% to 85.0%.^[5]^ The occurrence of gastrointestinal intolerance not only brings discomfort to patients but also can easily lead to the interruption of enteral nutrition, thus resulting in the failure of achieving the target supply.^[6–8]^

Enteral nutrition feeding intolerance is affected by many factors, including mechanical ventilation, sedative and analgesic drugs, disease, feeding mode, and feeding speed.^[9–12]^ Therefore, through the regulation of its influencing factors, the improvement of the tolerance of enteral nutrition is the key to the smooth implementation of enteral nutrition in critically ill patients. As feeding patterns are concerned, there are insufficient scientific evidences to prove which feeding method is safer and more reliable. However, there is no systematic review of the effects of intermittent feeding versus continuous feeding on enteral nutrition tolerance in critically ill patients. Therefore, this study objectively evaluated the efficacy and safety of intermittent feeding versus continuous feeding in enteral nutrition tolerance of critically ill patients, and provided scientific reference of nutritional support for critically ill patients.

## 2 Methods

### 2.1 Protocol register

This protocol of systematic review and meta-analysis has been drafted under the guidance of the preferred reporting items for systematic reviews and meta-analyses (PRISMA).^[13]^ Moreover, it has been registered on open science framework (OSF) (Registration number: DOI 10.17605/OSF.IO/4BP5X).

### 2.2 Ethics

As this study is an analysis of literatures, there is no need to recruit patients, and the privacy of patients will not be disclosed, so patients’ informed consent and ethical approval is not required.

### 2.3 Eligibility criteria

#### 2.3.1 Types of studies

We collected all available randomized controlled trials (RCTs) on intermittent feeding versus continuous feeding on enteral nutrition tolerance in critically ill patients, regardless of blinding, publication status, region, but languages are restricted to Chinese and English.

#### 2.3.2 Research objects

They include critically ill patients in intensive care unit; age ≥ 18 years; there is no contraindication of enteral nutrition, enteral nutrition starts from 24 to 28 hours, and patients need to receive enteral nutrition for more than 7 days.

#### 2.3.3 Interventional measures

When enteral nutrition was carried out, intermittent feeding was adopted in experimental group, and continuous feeding was applied in control group.

#### 2.3.4 Outcome indicators

(1) Gastric residue.
(2) Incidence of gastric retention.
(3) Incidence of abdominal distension.
(4) Incidence of vomiting.
(5) Incidence of diarrhea.

### 2.4 Exclusion criteria

(1) Duplicating published literatures, and choosing the one with the most complete data.
(2) If the paper is published as abstract or conference paper, the full-text paper cannot and the data cannot be obtained by contacting the corresponding author.
(3) Studies with obvious data errors.

### 2.5 Searching strategy

The combination of subject words and free words was searched in electronic databases, including WanFang, China National Knowledge Infrastructure (CNKI), China Science and Technology Journal Database, China Biology Medicine disc (CBM), Embase, PubMed, Web of Science, Cochrane Library, etc. RCT of intermittent feeding versus continuous feeding on enteral nutrition tolerance in critically ill patients was retrieved. Taking PubMed as an example, the retrieval strategy is displayed in Table 1.

**Table 1:** Retrieval strategy of PubMed.

Number	Search terms
1	Enteral Nutrition[MeSH]
2	Enteral Feeding[Title/Abstract]
3	Force Feeding[Title/Abstract]
4	Nutrition, Enteral[Title/Abstract]
5	Tube Feeding[Title/Abstract]
6	Gastric Feeding Tubes[Title/Abstract]
7	Feeding Tube, Gastric[Title/Abstract]
8	Feeding Tubes, Gastric[Title/Abstract]
9	Feeding, Enteral[Title/Abstract]
10	Feeding, Force[Title/Abstract]
11	Feeding, Tube[Title/Abstract]
12	Feedings, Force[Title/Abstract]
13	Force Feedings[Title/Abstract]
14	Gastric Feeding Tube[Title/Abstract]
15	Tube, Gastric Feeding[Title/Abstract]
16	Tubes, Gastric Feeding[Title/Abstract]
17	OR/1–16
18	Critical Illness[MeSH]
19	Critically Ill[Title/Abstract]
20	Critical Illnesses[Title/Abstract]
21	Illness, Critical[Title/Abstract]
22	Illnesses, Critical[Title/Abstract]
23	OR/18–22
24	Intermittent feeding[Title/Abstract]
25	Continuous feeding[Title/Abstract]
26	17AND 23 AND 24 AND 25

### 2.6 Data screening and extraction

**Figure 1:**
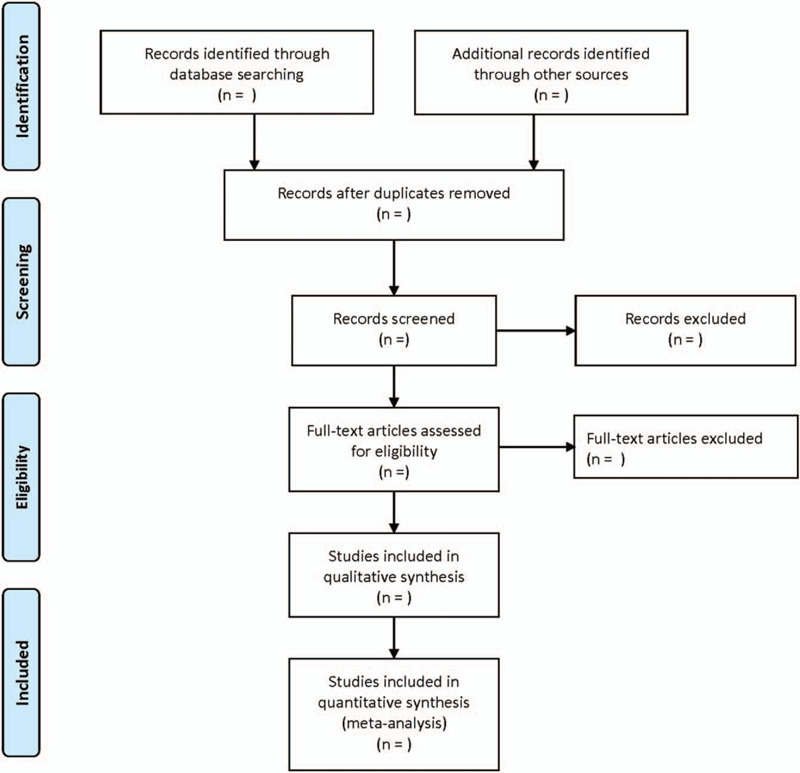
The process of literature screening.

First of all, 2 researchers independently browsed the literature title and abstract, and deleted the literature based on inclusion criteria and exclusion criteria. Then, the other 2 researchers read the full text of the remaining literatures after the exclusion of the title and abstract, listed tables to extract relevant data, excluded and screened, and finally included in the literature. Different opinions on literatures were discussed. If necessary, deciding whether to consult with the third researcher. Using the extraction table designed in advance to extract the research content, including basic data (title, author, publication date, source), research characteristics (number of cases, general demographic characteristics, intervention measures, follow-up, adverse events and so on), and indexes of outcome (gastric residue, incidence of gastric retention, incidence of abdominal distension, incidence of vomiting and incidence of diarrhea). The screening process is illustrated in Figure 1.

### 2.7 Literature quality evaluation

According to the bias risk assessment tool in Cochrane5.1.0, 2 researchers evaluated the authenticity of the literature. It includes 7 items: generation of random sequences, allocation hiding, blind method of subjects and researchers, blind method of outcome evaluator, incomplete outcome data, selective reporting, and other possible biases. Two researchers need to make low-risk, high-risk, and unclear judgment on each project, respectively. If the study fully meets these criteria, the possibility of various biases is small, and the quality grade is grade A. Some of the above quality standards are met, suggesting that the possibility of bias is moderate and the quality grade is grade B. If the above criteria are completely dissatisfied, the possibility of occurrence bias is high, and the quality grade is grade C.

### 2.8 Statistical analysis

RevMan 5.3 software was utilized for meta-analysis. The counting data were compared using relative risk (RR). The standard mean difference (SMD) is applied for continuity variable. All effect quantities are represented as 95% confidential interval (95% CI). First of all, χ^2^ test is carried out to determine whether the research problem has statistical heterogeneity. When *P* ≥ .1 and *I*^2^ <50%, it can be considered that the heterogeneity among multiple similar studies is acceptable, and the fixed effect model is selected to calculate combined statistics. If *P* < .1 and *I*^2^ ≥50%, it indicates that the heterogeneity of the research results is large. Therefore, subgroup analysis is needed. If there is no clinical heterogeneity and methodological heterogeneity, the random effect model is selected to calculate combined statistics. If it is impossible to detect the source of heterogeneity, meta-analysis is not performed and descriptive analysis is conducted.

#### 2.8.1 Dealing with missing data

If there are data missing in the article, the author needs to be contacted through email to get the relevant data. If the author cannot be contacted, or the author lost the relevant data, descriptive analysis will be conducted, without carrying out meta-analysis.

#### 2.8.2 Subgroup analysis

According to the type of enteral nutrition, the course of treatment, and the severity of the disease, we made a subgroup analysis.

#### 2.8.3 Sensitivity analysis

In order to ensure the stability of the outcome index results, the sensitivity analysis of each outcome index was performed.

#### 2.8.4 Assessment of reporting biases

When the number of articles with outcome index was greater than or equal to 10, the funnel chart is mapped to evaluate the publication bias.^[14–16]^ In addition, Egger and Begg test were conducted to evaluate potential publication bias.

## 3 Discussion

Most critically ill patients cannot eat by mouth, and lack nutrition, mainly due to the confusion of patients caused by serious diseases and the damage of gastrointestinal absorption function and so on.^[17]^ Therefore, nutritional support is an indispensable treatment for critically ill patients. At present, the combination of enteral nutrition and parenteral nutrition is recommended, and, when gastrointestinal function permits, enteral nutrition support is preferred.^[18–20]^

At present, the 2 methods of enteral nutrition in clinical application include both advantages and disadvantages. Intermittent feeding can establish a pattern of intermittent secretion of gastrointestinal hormones, which is more conducive to the establishment of a basic physiological environment for digestion and absorption in gastrointestinal tract. What is more, intermittent feeding can reduce the number of bacteria in stomach, especially at night.^[21]^ It can ensure the effective blood perfusion of gastrointestinal mucosa and prevent intestinal bacterial translocation,^[22]^ because the intragastric pH value is not affected by eating. However, another prospective control study proved that intermittent feeding without infusion pump has a higher incidence of gastric tube dislocation, aspiration pneumonia, and abdominal distension, compared with continuous infusion.^[8]^

The purpose of this study was to comprehensively evaluate the effects of intermittent feeding and continuous feeding on enteral nutrition tolerance and adequacy of critically ill patients based on Cochrane systematic evaluation method. However, due to the influence of the quantity and quality of included literatures, the evaluation of this system still has its limitations. Meanwhile, owing to the limitation of language ability, we only searched Chinese and English literatures, thus ignoring the study of other languages. Therefore, more high-quality randomized controlled trials are needed to further confirm the effectiveness and safety.

## Author contributions

**Literature retrieval:** Jin Yang.

**Data collection:** Shunxia Sun.

**Software operating:** Juan Huang and Aiguo Zhang

**Supervision:** Yuanli Li.

**Funding support:** Xiaoling Tang.

**Writing – original draft:** Yuanli Li and Xiaoling Tang.

**Writing – review & editing:** Yuanli Li and Xiaoling Tang.

**Data curation:** Shunxia Sun.

**Funding acquisition:** Xiaoling Tang.

**Methodology:** Jin Yang.

**Software:** Juan Huang, Aiguo Zhang.

**Supervision:** Yuanli Li.

**Writing – original draft:** Xiaoling Tang, Yuanli Li.

**Writing – review & editing:** Xiaoling Tang, Yuanli Li.
